# Commentary: It's tempting to jump down the rabbit hole

**DOI:** 10.1016/j.xjon.2020.05.013

**Published:** 2020-06-29

**Authors:** Ahmet Kilic

**Affiliations:** Division of Cardiac Surgery, Department of Surgery, Johns Hopkins University School of Medicine, Baltimore, Md

**Figure:**
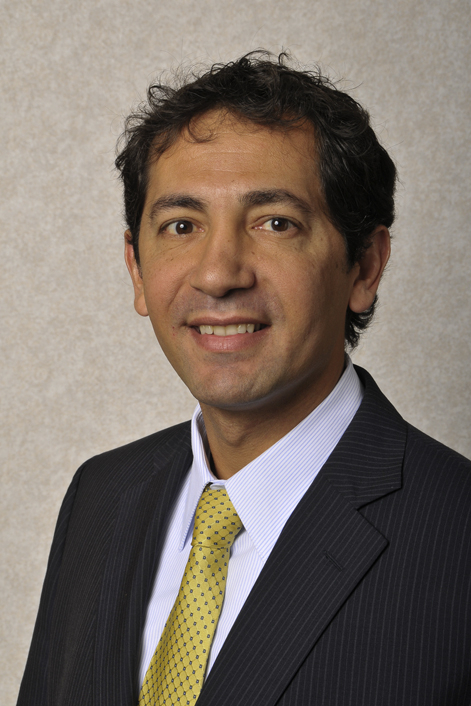
Ahmet Kilic, MD

**Central Message**

It is important to learn when not to use temporary mechanical circulatory support in futile endeavors.

See Article page 106.

“Good surgeons know how to operate, better ones when to operate, and the best when not to operate.”^1^

As surgeons, we are trained to be eternal optimists and to never give up on a patient; conversely, we are taught that a fatal complication can lurk in any nook and cranny. As we continue to push the boundaries of medicine and blur the lines between life and death, it becomes increasingly important to define what is and what isn't futility of care. As Rao and Billia^2^ aptly conclude in their article on when not to use temporary mechanical circulatory support (MCS), “the desire to prolong life can easily transform into an unintentional prolongation of death.”

The difficultly in this juxtaposition of life and death for these otherwise-moribund patients is that without MCS they will die and with MCS they have a shot at surviving. The subjectivity of hope and fear often drive the immediate step of erring on the side of initiating temporary MCS. The emotions surrounding death and that of hope are too strong of a pull, as is the nondecision of bridge to decision. In addition, the responsibility and task of being the ultimate judge and jury between life and death are daunting for those on the front lines. The algorithm outlined by the authors bears study—everything should start and be discussed in a shock team approach. It not only takes the onus of a life and death decision off of a sole person but also ensures that the subjectivity and emotions are kept in check. The underlying issues of reversibility and etiology of heart failure should be central and a pre-existing, institutional protocol adhered to as much as possible. If one thinks that not offering a patient MCS at a time of acute cardiac shock is challenging, the actual withdrawal of MCS in an otherwise-neurologically intact patient with no way out is absolutely demoralizing.

As we move more toward more percutaneous options for delivering biventricular support, it becomes instinctively easier to offer MCS. Despite the ease of use with noninvasive techniques, these devices do have significant medical costs and most importantly the same clinical dilemmas as we've outlined. The first step in any system should be to establish a team of specialists dedicated to making clinical decisions, outlining well-defined protocols, and a continuous feedback mechanism for all involved in the process.^3^ Conversations among the shock team should take into account the various elements outlined in the algorithm with a clarity and purpose of the next action steps. This does not need to take long, but 5 minutes of upfront concise, well thought-out, and decisive action will pay dividends moving forward.

Akin to the early days of heart transplantation, the prolongation of life via MCS is pushing the ethical boundaries of life and death.^4^ The central issue in this delicate balance remains the uncertainty in a chosen path—we should heed the advice of those who have traveled down the rabbit hole.
